# Infrared and Visible Image Fusion Technology and Application: A Review

**DOI:** 10.3390/s23020599

**Published:** 2023-01-04

**Authors:** Weihong Ma, Kun Wang, Jiawei Li, Simon X. Yang, Junfei Li, Lepeng Song, Qifeng Li

**Affiliations:** 1 Information Technology Research Center, Beijing Academy of Agriculture and Forestry Sciences, Beijing 100097, China; 2 School of Electrical Engineering, Chongqing University of Science & Technology, Chongqing 401331, China; 3 Advanced Robotics and Intelligent Systems Laboratory, School of Engineering, University of Guelph, Guelph, ON N1G 2W1, Canada

**Keywords:** infrared and visible light image, image fusion, evaluation index

## Abstract

The images acquired by a single visible light sensor are very susceptible to light conditions, weather changes, and other factors, while the images acquired by a single infrared light sensor generally have poor resolution, low contrast, low signal-to-noise ratio, and blurred visual effects. The fusion of visible and infrared light can avoid the disadvantages of two single sensors and, in fusing the advantages of both sensors, significantly improve the quality of the images. The fusion of infrared and visible images is widely used in agriculture, industry, medicine, and other fields. In this study, firstly, the architecture of mainstream infrared and visible image fusion technology and application was reviewed; secondly, the application status in robot vision, medical imaging, agricultural remote sensing, and industrial defect detection fields was discussed; thirdly, the evaluation indicators of the main image fusion methods were combined into the subjective evaluation and the objective evaluation, the properties of current mainstream technologies were then specifically analyzed and compared, and the outlook for image fusion was assessed; finally, infrared and visible image fusion was summarized. The results show that the definition and efficiency of the fused infrared and visible image had been improved significantly. However, there were still some problems, such as the poor accuracy of the fused image, and irretrievably lost pixels. There is a need to improve the adaptive design of the traditional algorithm parameters, to combine the innovation of the fusion algorithm and the optimization of the neural network, so as to further improve the image fusion accuracy, reduce noise interference, and improve the real-time performance of the algorithm.

## 1. Introduction

Visible images can provide the most intuitive details for computer vision tasks: however, due to the influence of the data acquisition environment, visible images do not highlight important targets [1]. Infrared images can compensate for the lack of visible light images [2]; therefore, image robustness can be improved by fusing infrared and visible light images [3]. After years of development, image fusion has matured: effective image fusion can extract and save important information from the image, without any inconsistencies in the output image, making the fused image more suitable for machine and human cognition [4].

Image fusion aims to create a fused image by integrating the dominant information from multiple images, thereby including more information through fusion [5]. There are six steps: image registration; feature extraction; decision marking; semantic equivalence; mapping calibration; and image fusion. During the image registration process, mapping of the source images is performed, matching equivalent images based on key features. To reduce computational costs, the image registration method aligns the subsequent features of different images with the reference image, exploiting the similarity. In feature extraction, multiple feature maps are generated by extracting important features from registered images. A set of decision maps is generated by labeling the registered images in terms of pixels or feature maps, using a decision operator. Semantic equivalence preserves decision or feature maps that may not be propagated to similar objects and are used to join these maps into a common object fusion. Map scaling is then applied to the spatially aligned images, to transform the feature maps at common scales to obtain a final result that is similar to the representative matrix. Finally, the fuser combines the corresponding images into a single image with improved image interpretation.

In recent years, in the field of image fusion, many methods have been proposed to fuse clear and fast images. Ma et al. [6] proposed a fusion algorithm based on gradient transfer and total variation minimization (GTF), and the fusion result resembled a sharpened infrared image with more details. Liu et al. [7] proposed an image fusion framework combining MST and SR to overcome the inherent shortcomings of MST and SRB-based fusion methods, and achieved state-of-the-art performance for the fusion of multimodal images. Li et al. [8] provided a deep learning architecture, in which the encoding network was combined with convolutional layers, fusion layers, and dense blocks, with the output of each layer connected to other layers. Li et al. [9] developed a novel end-to-end fusion network architecture (RFN-NEST) whose end-to-end fusion network outperformed state-of-the-art methods in the evaluation. Ma et al. [10] proposed a FusionGan generative adversarial network to fuse image information: this strategy produced clear fused images without the noise caused by the amplification of infrared information. Ma et al. [11] proposed Generative Adversarial Networks with Multi-Classification Constraints (GANMCC), which convert image fusion into a multi-distribution simultaneous estimation problem while retaining the fusion effect in the case of overexposed visible images. Zhang et al. [12] proposed an image fusion framework based on convolutional neural networks (IFCNN), and the proposed model outperformed existing models, in terms of generalization. Zhu et al. [13] proposed an attention-based deep neural network SDNET that used both attention and self-awareness to understand the dialogic environment and extract relevant information from paragraphs. Xu et al. [14] proposed a unified and unattended end-to-end image fusion network (U2Fusion) that addressed the problems of multimodality, multiple exposure, and multiple foci. Tang et al. [15] proposed a semantics-aware real-time image fusion network (SeAFusion). This method outperformed existing image fusion schemes in terms of maintaining pixel intensity distribution, preserving texture details, performance, and operational efficiency by cascading image fusion modules and semantic segmentation modules (cascading is a mapping relationship between multiple objects in computer science). These classical image fusion algorithms have solved some of the problems of traditional fusion algorithms, but these methods also have shortcomings, such as the long training time for image fusion models, the low efficiency of the fusion process, and the reduced pixel values of the fused images.

Currently, reviews of image fusion have focused mainly on fields of application, such as the medical field [16,17,18,19], the remote sensing field [20,21,22], and the image fusion index [23]; multisensor [24] and traditional image fusion methods have been summarized [25]. These overviews summarize image fusion technologies from many angles, each with its advantages and disadvantages; however, there are few reviews of fusion technologies for infrared and visible images. This paper took infrared and visible image fusion technology as its core, and divided them into traditional algorithms and deep learning technology, to establish application scope and an evaluation index for making a typical fusion technology comparison. Finally, the infrared and visible image fusion methods were summarized.

## 2. Research Status of Infrared and Visible Light Fusion Methods

The practicality of merging infrared and visible images has attracted the attention of many scholars. Several image fusion technologies have been proposed in recent decades, including traditional methods and methods based on deep learning [15]. The key to traditional image fusion algorithms lies in feature extraction and fusion. Multiscale decomposition is the most commonly used transformation method for feature extraction. Laplacian pyramid [26], wavelet transform [27], and multiscale geometric analysis [28,29,30] have been successfully embedded in the image fusion framework, based on multiscale transformation (MST). Sparse representation (SR) is used as a feature extraction technique, and the sparse base in the overcomplete dictionary is used to represent the source image [31]. In addition, subspace-based methods that project high-dimensional images into a low-dimensional subspace, to capture the internal structure of the source image, have attracted much attention. Deep learning technologies can be effectively applied to visible and infrared image fusion, which has the characteristics of fast fusion speed and clear fusion image. There are three commonly used networks, which are based on the Automatic Encoder (AE), the Convolution Neural Network (CNN), and the Generation Adversarial Network (GAN). 

### 2.1. Multiscale Transform

**Figure 1:**
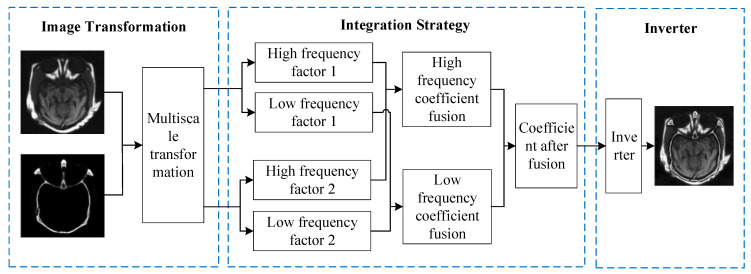
Multiscale transform-based image fusion framework.

The fusion process based on multiscale transform can be summarized as follows: (1) choose a multiscale decomposition method to decompose the image separately, in order to obtain high-frequency sub-bands and low-frequency sub-bands at different frequency levels; (2) design the optimal fusion calculation method as the fusion strategy, according to the different characteristics of the high-frequency sub-bands and low-frequency sub-bands, and perform the fusion operation on the coefficients of the high-frequency sub-bands and the low-frequency sub-bands, respectively; (3) invert the final fusion coefficients, to generate the fused image. The multiscale transform-based image fusion method can design a suitable fusion strategy according to the characteristics of different sub-bands, and the fused image is rich in detailed information and low in redundancy. The framework of multiscale transform-based image fusion is shown in Figure 1. The selection of the decomposition method and fusion rules is a key part of the fusion process, which determines whether the fused image can retain more information than the original image: it mainly includes pyramid transform, wavelet transform, and geometric transform without subsampling in multiple scales and multiple directions. Table 1 shows the advantages and disadvantages of the multiscale decomposition method.

Some scholars have addressed the existence of multiscale fusion transformations’ low image contrast, information redundancy, low robustness, serious loss of source image structure information, and low image signal-to-noise ratio. False color fusion based on color reference images [39] and fuzzy logic with superb edge representation, to enhance the fusion effect of LP-transformed images, is proposed. It has also been proposed that directional filters can be combined to solve the directional invariance problem of the CP transform [40]. Each scale in the wavelet transform has high independence and high retention of texture edge information [41]; however, DWT has some defects, including oscillation, shift error, aliasing, and lack of directional selectivity [42]. DT-DWT solves the problem that DWT lacks directionality: DT-DWT has less redundant information, and has high computational efficiency. Lifting wavelet transform has the advantages of strong adaptive design and irregular sampling, and the fusion visual effect is better [43]. Multiscale geometric analysis solves the spectral mixing problem of the contour wave transform. To address the defects of the multiscale geometric analysis method, some scholars have proposed a combination of NSCT and fuzzy logic, to effectively enhance infrared targets and preserve the details of visible images [44]. The combination of NSCT and an extracted target area can successfully highlight infrared targets [45]. Guo et al. [46] have proposed a multiscale and multidirectional shear wave transform, which meets the demand for high real-time performance. NSST has higher computational efficiency than NSCT. Kong et al. [47] have proposed the fast non-negative matrix factorization in the NSCT fusion method, which reduces redundant information in images.

### 2.2. Sparse Representation

Sparse representation [48] expresses most or all of the original signal with a linear combination of fewer basic signals. The image fusion method based on sparse representation is generally divided into four steps, and we take multispectral images (MS) as an example, as follows:

(1) Construct a linear regression model of the MS image and luminance components.

The luminance is defined using adaptive weight coefficients. The panchromatic image is resampled downward, to obtain a panchromatic image with the same spatial resolution as the MS image, and then the least binary method is used to solve equation (1), to obtain the weight coefficients $g_{b}$ and the bias constant $\beta_{\mathrm{bias}}$. The resulting linear relationship is used to simulate the luminance component of the low-resolution MS image I, and the panchromatic image is histogram-matched with the I image to obtain the image $M_{P}^{\prime }$, which has similar mean and variance to I.

(1) $$I=\sum_{b=1}^{B}g_{b}M_{\mathrm{MS},b}+\beta_{\mathrm{bias}}$$

where *B* is the number of bands, $M_{\mathrm{MS},b}$ denotes the *b*-band image of the original MS image, and the weight coefficient, $g_{b}$, and the bias constant, $\beta_{\mathrm{bias}}$, are obtained by solving the following linear regression problem by the least binary method:

(2) $$M_{P}^{l}=\sum_{b=1}^{B}g_{b}M_{\mathrm{MS},b}+\beta_{\mathrm{bias}}$$

where $M_{P}^{l}$ is the degraded panchromatic image.

(2) Sparse representation process.

The MS image [49] is resampled to the same size as the panchromatic image and denoted as $M_{\mathrm{MS}}^{l}$. A window of size $\sqrt{n}\times \sqrt{n}$ is used to traverse each band of the MS image and the full-color image from left to right and from top to bottom, and each image block is converted into a column vector of length *n*, denoted as ${\left\{x^{M_{\mathrm{MS},b}^{l}}\right\}}_{i=1}^{N},{\left\{x_{i}^{M_{P}^{\prime }}\right\}}_{i=1}^{N}$, where *N* is the number of image blocks in a single image and $M_{\mathrm{MS},b}^{l}$ denotes the *b*th band of the MS image $M_{\mathrm{MS}}^{l}$. $M_{\mathrm{MS},b}^{l}$, $M_{P}^{\prime }$, solved by Equations (3) and (4).

(3) $$\alpha_{M_{\mathrm{MS},b}^{l}}=\arg\underset{\alpha_{M_{\mathrm{MS},b}^{l}}}{\min}\left\|\alpha_{M_{\mathrm{MS},b}^{l}}\right\|{}_{0}\;\;\;\;\;s.t.\;\;{\left\|x_{i}^{M_{\mathrm{MS},b}^{l}}-D_{l}\alpha_{M_{\mathrm{MS},b}^{l}}\right\|}_{2}^{2}⩽\varepsilon$$

(4) $$\alpha_{M_{p}^{\prime }}=\arg\underset{\alpha_{M_{p}^{\prime }}}{\min}\alpha_{M_{p}^{\prime }0}\;\;\;\;\;s.t.\;\;{\left\|x_{i}^{M_{p}^{\prime }}-D_{h}\alpha_{M_{p}^{\prime }}\right\|}_{2}^{2}⩽\varepsilon$$

As the sparse representation coefficient corresponds to the atoms in the dictionary, the magnitude of the sparse representation coefficient reflects the degree of significance of the corresponding atoms; therefore, the sparse representation coefficient of the low-resolution luminance image is:

(5) $$\alpha_{I_{0}}=\sum_{b=1}^{B}g_{b}\alpha_{M_{\mathrm{MS},b}^{l}}+\beta_{\mathrm{bias}}$$

(3) Detailed information injection.

The sparse representation coefficients of the panchromatic images are partially replaced by the absolute maximum fusion rule, to obtain the sparse representation coefficients of the high-resolution luminance components:

(6) $$\alpha_{I}(i)=\left\{\begin{array}{cc} \alpha_{M_{P}}(i) & \left|\alpha_{M_{P}}(i)\right|>\left|\alpha_{I_{0}}(i)\right|\\ \alpha_{I_{0}}(i) & \mathrm{other} \end{array}\right.$$

where $\alpha_{I}$ denotes the sparse representation coefficient corresponding to the high-resolution luminance component, and *i* denotes the *i*th element of the sparse representation coefficient.

Thus, according to the component replacement fusion framework, the sparse representation coefficients corresponding to the high-resolution MS image can be obtained as:

(7) $$\alpha_{M_{\mathrm{MS},b}}^{h}=\alpha_{M_{\mathrm{MS},b}^{l}}+w_{b}\left(\alpha_{I}-\alpha_{I_{0}}\right)$$

where $w_{b}$, denotes the weight coefficient corresponding to the *b*th band, defined as $w_{b}=\frac{\mathrm{cov}\left(I,M_{\mathrm{MS},b}\right)}{\mathrm{var}(I)}$, *I* is the luminance component extracted in step (1), and $M_{\mathrm{MS},b}$ is the *b*th band of the original low-spatial resolution MS image.

(4) Image

**Figure 2:**
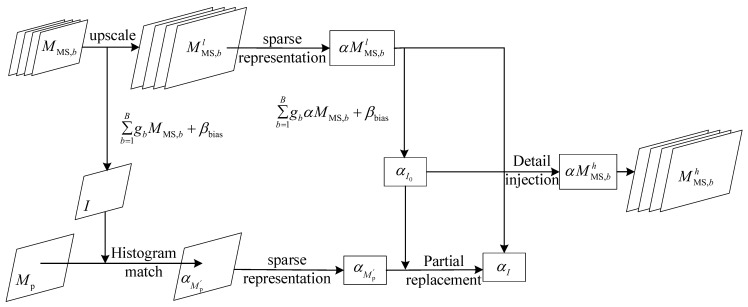
Flow chart of image fusion algorithm based on sparse representation.

According to $x=D_{h}\alpha$, can be reconstructed from the resulting sparse representation coefficients, into a high-resolution MS image. The flow of the image fusion algorithm based on sparse representation is shown in Figure 2.

Compared to traditional multiscale transform, sparse representation has two main differences [50]: firstly, the multiscale fusion method is based on a pre-set basis function, which makes it easy to ignore some important features of the source image, while sparse representation learns an over-complete dictionary, which can better express and extract images; secondly, the multiscale transform-based fusion method uses multiscale methods to decompose images into multi-layer images, but as the number of decomposition layers increases, the requirements for image fusion in terms of noise and registration become more and more stringent. The sparse representation uses a sliding window technique to segment the image into multiple overlapping patches, which are then vectorized to reduce image artifacts and to improve robustness against misregistration. Although the image fusion method based on sparse representation can improve the problems of insufficient feature information and high registration requirements in a multiscale transformation, it still has some shortcomings, which are mainly reflected in three aspects: firstly, the signal representation capability of the overcomplete dictionary is limited, which can easily lead to the loss of image texture detail; secondly, the Max-L1 fusion rule is sensitive to random noise, which reduces the signal-to-noise ratio of the fused image; thirdly, there is the overlapping small block segmented by the sliding window technology, which reduces the operational efficiency of the algorithm.

### 2.3. Subspace-Based

In image fusion problems, subspace learning is a relatively common method. By learning a suitable subspace, images that are not easily identified or distinguished in the original space are expanded in the subspace, or the subspace has some advantages that the original space does not have, and then the samples are mapped to the subspace to obtain better classification results. As visual features in zero-sample image classification are extracted by neural networks, while semantic features are obtained by manually defined attributes or keywords extracted from text, the distributions of visual and semantic features are usually different. If the mapping between visual space and semantic space is obtained by direct learning, the knowledge transfer ability is usually not strong, resulting in poor performance of zero-sample recognition. Through the learning of subspace, the alignment between semantic space and visual space can be achieved, and better knowledge transfer capability can be obtained.

In this method, the high-dimensional input image is projected into the low-dimensional space or subspace to capture the internal structure of the original image [51]. Typical subspace-based methods include principal component analysis (PCA) [52], independent component analysis (ICA) [53], and non-negative matrix factorization (NMF) [54]. PCA converts related variables into unrelated variables, and preserves the information of the original data while reducing the dimensions [55]. Bavirisetti et al. [56] decomposed the source image into approximation and detail images, using the image decomposition method, then fused the detail images with PCA, and the approximation images with averaging rules, and finally reconstructed the image by combining the approximation and detail images. As an extension of PCA, ICA-based methods typically use multiple natural images with similar content, to train a set of bases that can be fused to images with similar content. Cvejic et al. [57] proposed a region-based ICA fusion method. This method divides the image into multiple regions, and then extracts ICA coefficients from each region, using the pre-processed image. According to the fusion image quality maximization criterion, the Piella fusion metric is used to weigh ICA coefficients. NMF is a component-based object representation model [58] that decomposes the source data matrix into the product of two non-negative matrices. Mou et al. [59] proposed a fusion method, combining NMF and infrared target extraction, that uses NMF to preserve the global features of infrared and visible images.

### 2.4. Automatic Encoder

**Figure 3:**
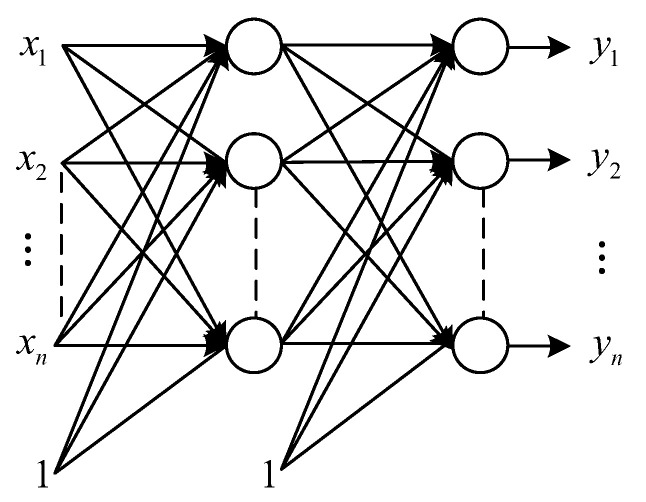
Schematic diagram of AE network structure.

AE can convert high-dimensional data into low-dimensional representation, and it is a three-layer network containing an input layer, a hidden layer, and an output layer. The network structure is shown in Figure 3, where the hidden layer has *m* nodes, the output layer and the inflow layer both have *n* nodes, and 1 is the bias amount, where the input to the network is represented as $x=\left(x_{1},x_{2},\cdots ,x_{n}\right)$, and the output is expressed as $y=\left(y_{1},y_{2},\cdots ,y_{n}\right)$. Therefore, the global cost function of AE is shown in Equation (8), and the single cost function is shown in Equation (9):

(8) $$E=\frac{1}{2}\sum_{i=1}^{s}\sum_{j=1}^{n}{\left(y_{j}^{i}-x_{j}^{i}\right)}^{2}$$

(9) $$E_{l}=\frac{1}{2}\sum_{j=1}^{n}{\left(y_{j}-x_{j}\right)}^{2}$$

where: s is the number of input samples, n is the input dimension, $x_{j}^{i}$ denotes the j component of the sample i, and $y_{j}^{i}$ denotes the j component of the output corresponding to the sample i. When the output error E is small enough, it means that the input sample data can be reconstructed by the hidden layer, and then the output of the hidden layer is the extracted sample features.

The AE framework is an important branch of machine learning that trains an automatic encoder to realize feature extraction and reconstruction. Li et al. [60] proposed a simple fusion architecture comprising three parts: the encoder layer, the fusion layer, and the decoder layer. The encoder layer contains a convolutional layer and dense blocks with high-level features, where the dense blocks are used in the encoding process to get more useful features. In the fusion layer, the element addition strategy or l1-norm strategy is used to merge high-level features, and the feature reconstruction network includes four convolutional layers to reconstruct the fused image. In addition, Li et al. also introduced a multiscale encoder-decoder architecture and nest connection [61], to extract richer features. However, the above methods use hand-made fusion rules to integrate depth features, which severely limits fusion performance. To solve the limitations of hand-designed fusion rules, Xu et al. [62] proposed a saliency-based classification rule for the AE-based image fusion framework. This new fusion rule uses a classifier to measure the magnitude of each pixel in the feature map, and calculates the fusion weight according to each pixel’s contribution.

### 2.5. Convolution Neural Network

**Figure 4:**
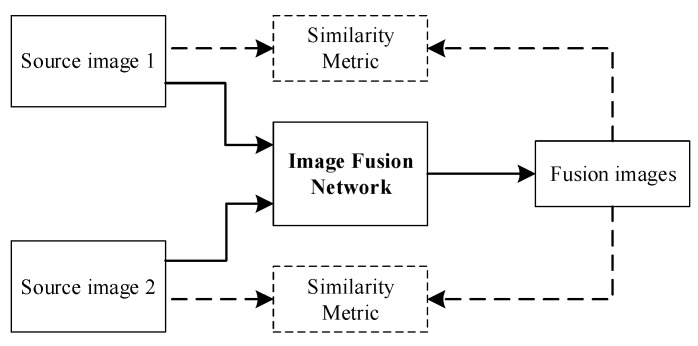
CNN-based image fusion framework.

CNN is widely used in the field of image recognition: it is a kind of artificial neural network, and the structure of CNN can be divided into three layers: convolutional layer, pooling layer, and fully connected layer. The convolutional layer is used to find features, and then the fully connected layer is used to make classification judgments, while the pooling layer is used to allow training with fewer parameters, and to ignore some information while keeping the sampling constant. The CNN-based image fusion framework is shown in Figure 4.

The CNN-based fusion framework either realizes hidden feature extraction, aggregation, and image reconstruction under the guidance of a carefully designed loss function or uses CNN as part of the overall fusion framework to realize activity-level measurement and feature integration. LP–CNN is a pioneer in the use of CNN in image fusion, combining LP with classified CNN to achieve medical image fusion [63]. In addition, Zhang et al. [12] developed a general image fusion framework through a general network structure, namely the feature extraction layer, the fusion layer, and the image reconstruction layer. The fusion layer is embedded in the training process: as a result, IFCNN can alleviate the constraints imposed by artificially designed fusion rules (element maximum, element minimum, or element average).

In addition, the researchers also studied another solution: a CNN-based end-to-end image fusion framework, to avoid the shortcomings of hand-made rules. The CNN-based method inherited the core concept of the traditional optimization-based method, which defines the objective function of image fusion as overall intensity fidelity and preservation of texture structure [6]. Zhang et al. [64] modeled uniform image fusion as proportional preservation of gradient and intensity, and designed a general loss function for various image fusion tasks. Based on the gradient and intensity path, they also designed an extrusion and decomposition network, to improve the fidelity of fused images [65]. Additionally, an adaptive decision block was introduced, to assign the weight of gradient loss elements according to the texture richness of the source image. Considering the cross-fusion between different image fusion tasks, Xu et al. [14] trained a unified model for multiple fusion tasks. To improve the semantic information in the fused image, Ma et al. [66] used a highlight mask to construct the necessary information for the fusion of infrared and visible images. Although the proposed network could detect salient targets, the simple salient target mask only enhanced the semantic information of the salient target area. In addition, for image fusion tasks, it was difficult to provide the ground truth to construct the loss function, which meant that the CNN-based fusion network could not fully unlock its potential power.

### 2.6. Generate Adversarial Network

GAN designs the generative model as a model to learn probabilistic parameters. In order to minimize the scatter between the real distributed data and the generative model, two models are trained simultaneously to estimate the generative model through a minimum–maximum game adversarial process: the generative model *G* and the discriminative model *D*. The generator *G* takes one of the generated samples, to deceive the discriminator *D*, which distinguishes between real and fake images. Adversarial learning is performed during the training process, to improve the performance of both models and to produce higher-quality images. Its objective function is:

(10) $$\underset{G}{\min}\underset{D}{\max}V(D,G)=E_{x∼P_{\mathrm{data}}}[\log D(x)]+E_{x∼P_{G}}[\log(1-D(x))]$$

**Figure 5:**
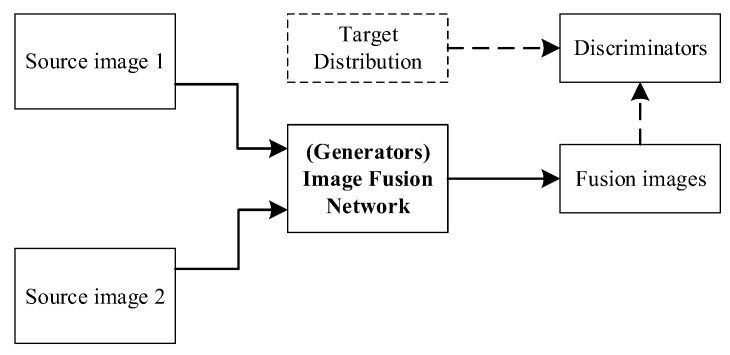
GAN-based image fusion framework.

The parameters in discriminator *D* are constant when training *G*. The adversarial process between *G* and *D* constitutes a minimum–maximum game, where *G* tries to fool *D*, and *D* is trained to discriminate the generated data; therefore, it is difficult for the discriminator to distinguish the generated samples from the real data. The existing GAN-based fusion methods only apply GAN to force the fused image to obtain more details in the visible image, while the thermal radiation in the infrared image is only obtained by content loss. As the adversarial game proceeds, the fused image becomes more similar to the visible image, and the prominence of the thermal target gradually decreases. The above problems can be solved by using dual discriminators. The GAN-based image fusion framework is shown in Figure 5.

As antagonistic loss is constructed from a probability distribution perspective, an antagonistic generative network is an ideal choice for unsupervised tasks such as image-to-image translation [67,68] and image fusion [69]. Ma et al. [10] creatively introduced the generation countermeasure network to the field of image fusion, forcing the generator to synthesize fused images with rich textures using discriminators. To improve the quality-of-detail information and sharpen the edges of thermal targets, they also introduced loss of detail and edge-enhancement loss [70]. However, a single discriminator can lead to a pattern in the fused image that is biased toward visible or infrared images; therefore, Ma et al. [71] further proposed a double discriminator conditional generation countermeasure network, to improve the robustness of the GAN-based framework and to maintain the balance between infrared and visible images. Subsequently, Li et al. [72] integrated a multiscale attentional mechanism into the GAN-based fusion framework, causing the generator and discriminator to pay more attention to typical regions. Furthermore, Ma et al. [11] transformed image fusion into a multi-distribution synchronization estimation problem and realized the classifiers’ balance between infrared and visible images. Traditional methods and deep learning methods, on the other hand, emphasize the improvement of fused image quality and evaluation indicators, while ignoring the needs of high-level visual tasks. In practice, fused images with excellent image quality may be suitable for human visual perception but may not encourage demanding visual tasks. An efficient image fusion algorithm should fully integrate the complementary information of the source image, and enhance the semantic information of the fused image.

### 2.7. Hybrid Model

Mixed models can improve image fusion performance by combining the advantages of different methods. Common mixed models include multiscale transformation and saliency detection, multiscale transformation and SR, multiscale transformation and PCNN. Image fusion methods that combine multiscale transformation and expression detection are generally integrated into the fusion framework of multiscale transformation to improve the image information of the area of interest. Saliency detection has two main application methods: weight calculation [73] and salient target extraction [74]. The weight calculation consists in obtaining a saliency map in high- and low-frequency sub-band images, calculating the corresponding weight map, and finally applying it to the image reconstruction. Significant target extraction is often used in surveillance applications, such as target detection and recognition. Zhang et al. [75] used saliency analysis to extract target information from infrared images based on the NSST fusion framework.

The multiscale transformation has the problems of low image contrast and difficult determination of the multiscale decomposition level. The sparse representation shows that the source image’s texture and edge information tend to be smooth and that the computational efficiency is low. In combination with multiscale transformation and sparse representation, the hybrid model can usually achieve the best balance. The sparse representation model is usually applied to low-frequency sub-band images after multiscale decomposition [76]. Additionally, due to the advantage that PCNN can fully extract image detail information, multiscale transform is often combined with SR and PCNN, and the fusion rules based on SR are selected at low frequencies and PCNN at high frequencies [77]. The hybrid model effectively improves the clarity and texture features of the fused image, but when designing the fusion model, the advantages and disadvantages of SR and PCNN must be coordinated, to avoid model complexity and increased computational cost.

## 3. Application of Image Fusion Technology in Different Fields

In recent years, image fusion has been widely used in many fields, such as robotic vision, medical imaging, remote sensing [78], and telemetry: image fusion plays an important role in the pre-processing phase of these areas [79]; this section discusses different challenges and problems in different areas.

### 3.1. Robot Vision Field

**Figure 6:**
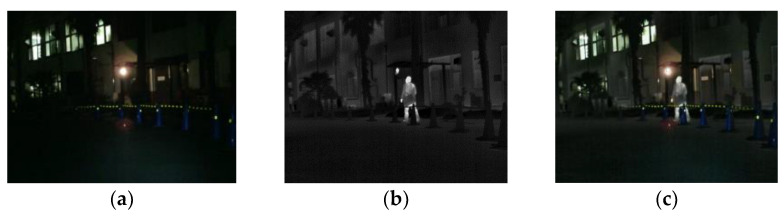
Image fusion in the robot vision field: (**a**) visible light map; (**b**) infrared image; (**c**) fusion image.

The fusion of infrared and visible images is widely used in the robotic detection of living objects. Infrared images distinguish the target from the background according to the difference in thermal radiation, and are not affected by illumination and weather conditions; however, infrared images cannot provide texture detail. Visible light images can provide the most intuitive detail for computer vision tasks; however, due to the influence of the data collection environment, visible images may not highlight important targets. Infrared and visible light fusion images can solve the shortcomings of a single image, to extract information. Figure 6 shows an example of the fusion of infrared and visible images. In the fused image, we can see the unrecognizable information under the visible light image, and the fused image is clearer than the infrared image. The processed fused image can also make the computer recognize, train and process better. In addition, the fusion of visible and infrared images has also been introduced, such as intelligent animal husbandry, automatic driving, and face recognition.

Currently, the main challenges in this area are computational efficiency: an effective image fusion algorithm should innovatively fuse image information to obtain the final image. Also, real-time surveillance in these areas generates a large amount of image information, which requires high computational efficiency to process this information. The main difficulty in this field is that images may be obtained in imperfect conditions, e.g., due to weather and lighting conditions, the input image may contain underexposure and excessive noise.

### 3.2. Field of Medical Imaging

**Figure 7:**
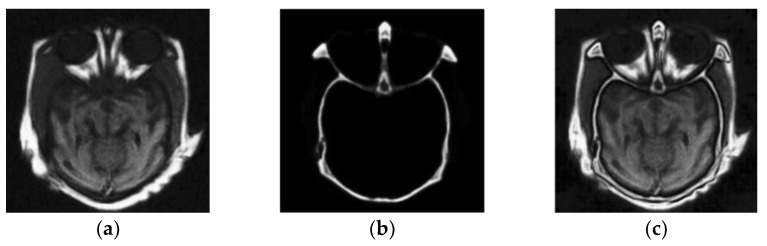
Image fusion in the field of clinical imaging: (**a**) MRI; (**b**) CT; (**c**) fusion image.

Image fusion is also widely used in the field of medical imaging. At present, the medical imaging mode generates various types of medical images to help doctors diagnose diseases or injuries. Each form of an image has its specific intensity. Many medical imaging researchers tend to combine redundant information and related information from different medical images, to create fused medical images that provide additional centralized and information-inspired image diagnosis for the medical examination. Figure 7 shows an example of image fusion for medical diagnostics by merging CT and MRI. The data comes from a brain image dataset composed of computed tomography and magnetic resonance imaging provided by Harvard Medical School. CT is used to capture bone structures with the high-spatial resolution, and MRI is used to capture soft tissue structures such as the heart, eyes, and brain. CT and MRI can be used together with image fusion technology to improve accuracy and reasonable medical applicability.

The challenges in this field are as follows: 1. Lack of medical crisis-oriented image fusion methods: the main motivation of image fusion is to help improve clinical outcomes; clinical crisis continues to be a major challenge in the medical field; 2. Objective evaluation of image fusion performance: the main difficulty in this area is how to evaluate image fusion performance; there are many clinical issues with image fusion, one of which is that the fusion effects of different procedures can vary widely; 3. Incorrect registration: in the medical field, inaccurate registration of objects leads to poor performance.

### 3.3. Agricultural Remote Sensing Field

**Figure 8:**
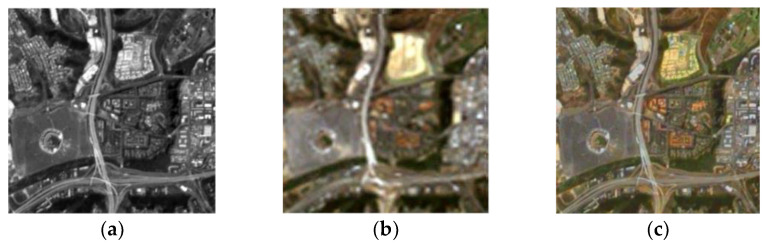
Image fusion in the field of agricultural remote sensing: (**a**) PAN; (**b**) MS; (**c**) fusion image.

Image fusion technology is also widely used in the field of agricultural remote sensing. Based on agricultural remote sensing technology, the selection of the environment for the adaptation of plants and the detection of plant diseases can be carried out. Existing fusion technologies, including equipment such as the synthetic aperture radar, ranging and optical detection, and medium-resolution imaging spectrometers, all have applications in image fusion. Byun et. al. [4] presented a region-based fusion scheme for combining panchromatic, multispectral, and synthetic aperture radar images. Temporal data fusion and high spatial methods were used to generate synthetic Landsat imagery by combining Landsat and Moderate Resolution Imaging Spectrometer data. In addition, the combination of spectral information, optical detection, and radar range data has recently been studied. Various datasets provided by Earth-imaging satellites, such as Quickbird, Worldview-2, and IKONOS, have been used for pan-sharpening applications. Acquiring simultaneously registered hyperspectral and multispectral images is more complicated, compared to multispectral and panchromatic images. In addition, it is not difficult to obtain hyperspectral data and radar data from gas bones. For example, the 2013 and 2015 IEEE Society for Geosciences and Remote Sensing data fusion competitions published a large amount of hyperspectral, color, and optical detection and range data for research purposes. Figure 8 shows an example of image fusion in the field of agricultural remote sensing. Many satellites were used to obtain remote sensing images with different spatial, temporal, and spectral resolutions. In addition, the classification and change detection of Google Maps or other mapping products has been provided in this area, effectively applied to create images. Compared to pan-sharpening, multichannel multispectral images contain both spatial and spectral information.

Currently, this field faces the following challenges: 1. Spatial and spectral distortion: image datasets often exhibit changes in spatial and spectral structures that result in increased spatial or spectral distortion in the image fusion process; 2. Misregistration: the most important challenge in this area is to reduce the misregistration rate. Remote sensing input images are often obtained from different time, acquisition, or spectral bands. Even with panchromatic and multispectral datasets provided by similar platforms, one or more sensors cannot provide accurate results in the same direction, and can have different gain times: to solve this problem, it is necessary to register the images before image fusion. In addition, registration is a challenging process, because the input images are provided by different collections, and there are differences between them.

### 3.4. Industrial Defect Detection Field

Due to the constraints of industrial production conditions, workpiece defects [80] are difficult to avoid. Typical defects include slag, porosity, and cracks inside the workpiece. These defects evolve during the use of the workpiece, and affect the performance of the workpiece, eventually causing the workpiece to fail, shortening its service life, and threatening the safety of the machine. The clearer the shape, area, and location of defects in the workpiece, the more accurate the reliability assessment of the workpiece will be.

**Figure 9:**
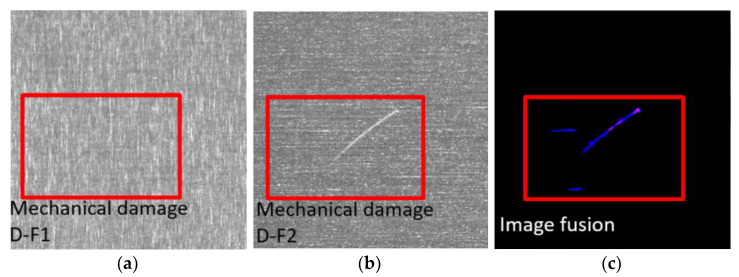
Image fusion in the field of industrial defect detection: (**a**) Image1; (**b**) Image2; (**c**) fusion image (The red box is the area of mechanical damage, which is used to emphasize the obvious fusion effect of the images).

The current defect detection algorithm is generally divided into two steps: (1) defect area segmentation, where all potential defect areas are segmented from the image, and the area with closed boundaries is selected as the defect candidate area; (2) candidate area discrimination, where the segmented candidate area is screened, based on shape features, grayscale features, and Hu-invariant moment “features”. This part of candidate region discrimination requires human participation in designing some main features of the defects, which presents a problem: the manually designed features are not very robust against changes in the diversity of defects, and are only applicable to specific defect detection, which is difficult to adapt to automatic recognition [81] and localization of images with different sizes of defects, diverse shapes, and complex background areas. Figure 9 shows an example of image fusion in the field of industrial defect detection [82]:

## 4. Main Evaluation Indexes of Image Fusion

To assess the quality of fused infrared and VI images, most researchers often use the image fusion assessment index [83], which can be divided into subjective assessment and objective assessment. The purpose of the image quality assessment method is to measure the contribution of the source image to the fusion image, and it can also be used to find the optimal setting of the parameters of a particular fusion algorithm [84]. In addition, the fusion image evaluation method can be used to evaluate the effectiveness of the image fusion method [85].

### 4.1. Subjective Evaluation

Subjective rating is the most commonly used and direct method of assessing the quality of fused images from the perspective of human vision, because the end user and the interpreter of the fused image are human, which makes the subjective evaluation method very important in IR and VI image fusion [86]. The subjective rating is divided into an absolute rating and a relative rating, which are evaluated using a recognized five-point quality scale and an obstacle scale, respectively: this is used to assess image definition, edge definition, and image distortion level, and to retain the level of detail of the source image. The subjective evaluation of the fused images is based on the subjective judgment of the human eye, which has a certain one-sidedness and randomness; therefore, the quality assessment of the fused images must be analyzed comprehensively, and compared with the objective assessment [60].

### 4.2. Objective Evaluation

The objective evaluation calculates the relevant index information of the image, using a specific formula to quantitatively analyze the fused image. Although it is used to evaluate the performance of infrared and visible image fusion algorithms, some of the evaluation metrics for others, such as multi-exposure, multi-focus, and medical image fusion, are also applicable. This paper has organized and summarized some of the currently available evaluation metrics. A total of 17 evaluation methods were organized. The performance evaluation metrics were mainly divided into four categories, which were: information-theory-based evaluation metrics, structural similarity-based evaluation metrics, structural similarity-based evaluation metrics, and evaluation metrics based on source image and generated image, Among them: information-theory-based evaluation metrics, mainly including information entropy (EN), [87] mutual information (MI), pixel feature mutual information (FMI_pixel), discrete cosine feature mutual information (FMI_w), wavelet feature mutual information (FIM_dct), and peak signal-to-noise ratio (PSNR); structural similarity-based evaluation metrics, mainly including structural similarity measure (SSIM), multiscale structural similarity measure (MS_SSIM), and mean square error (MSE); evaluation metrics based on image features, mainly including spatial frequency (SF), standard deviation (SD), and average gradient (AG); evaluation metrics based on human visual perception, mainly including visual fidelity (VIF) [88]; evaluation metrics based on source image and generated image, mainly including correlation coefficient (CC), sum of difference correlation (SCD), gradient-based fusion performance (Qabf) [89], and noise-based evaluation of fusion performance (Nabf). Image fusion evaluation metrics are complicated, and some of them are similar, so we selected typical examples of these evaluation methods, to do a detailed explanation, as shown in Table 2:

EN can reflect the average information from the fused image, and represent the texture richness of the image. The larger the EN, the richer the information from the fused image. MI measures the degree of similarity between the two images, i.e., how much information from the original image is acquired by the fused image. The greater the mutual information, the better the quality of the fused image, as it retains more information from the source image. VIF is used to quantify image distortion, including additional noise, blur, and global or local contrast. SF denotes image detail clarity and spatial variation. The larger the SF, the richer the texture and edges. SF is also independent of the reference image. SD is used to evaluate the deviation between pixel and pixel mean. With the increase in SD, the SD also increases; thus, improving the contrast of the image. Qabf is an objective non-reference quality assessment metric for fused images. The algorithm of Qabf uses a local metric to estimate how well the significant information from the input is represented in the fused image, and a higher value of Qabf indicates the better quality of the fused image.

The evaluation index based on the source image usually takes into account only a particular statistical feature of the image, unlike the subjective evaluation results, which mainly measure the information extracted from the source image from an information-theoretic perspective. EN is one of the most commonly used indexes for evaluating image quality; however, when there are artifacts and noise in the fused image, the value of EN increases sharply, causing the evaluation results representing the final image quality to fail. AG evaluates the fusion effect only through the fused infrared and VI images and does not rely on the standard reference image. AG and SF reflect the grayscale rate of change and sharpness of the image, and the artifacts of SF in the fused IR and VI images multiply the value of SF. In this case, SF cannot correctly reflect the quality of the fused image. In addition, MI, CC, and Qabf edge information transmission are commonly used. MI describes the amount of information from the source image that is fused into the final image: the larger the MI, the more information from the source image to the final image, which means the image fusion method is more effective. The range Qabf is [0, 1], and the closer the value is to 0, the more edge information is lost. Conversely, the closer the value is to 1, the more information is stored. In addition, there is cross-entropy and common entropy derived from information entropy. EN only reflects the information of the fused image, which cannot explain the overall fusion effect of the image; however, cross-entropy and shared entropy can make up for this deficiency. 

The evaluation index based on the reference image aims to evaluate its performance by comparing the difference in gray value and noise between the source image and the standard reference image. SSIM evaluates the performance of images by comparing the differences in brightness, contrast, and textural distortion values between images. RMSE evaluates quality by comparing pixel gray values between images; peak signal-to-noise ratio (PSNR) represents the ratio of the maximum possible power of a signal to the power of distortion noise that affects the quality of its effectiveness and is used to measure the proximity of the source image to the final image.

In the actual image fusion process, there is often no standard reference image, so this evaluation method is not yet used on a large scale. A single rating index cannot effectively represent the quality of the result; therefore, various objective evaluation indicators are used together to measure how much information is fused from the source image into the final image.

## 5. Qualitative and Quantitative Testing of Mainstream Image Fusion Technology

We selected infrared and visible images from four application scenarios and used 10 representative fusion methods and six score indices for comparative experimental analysis. To fully assess the fusion performance of this method, we compared these 10 algorithms, based on the MFNet dataset.

### 5.1. Qualitative Results

The MFNet dataset contains two typical scenarios: the daytime scenario and the night-time scenario. To show the advantages of the fusion framework for fusing complementary information and improving the visual quality of fused images, we selected two daytime scenes and two night-time scenes for subjective evaluation. For daytime scenes, the thermal radiation information of the infrared image could be utilized as complementary information to the visible image. A good fused image should contain the rich textural details of the visible image, and enhance the prominent targets in the IR image. 

**Figure 10:**
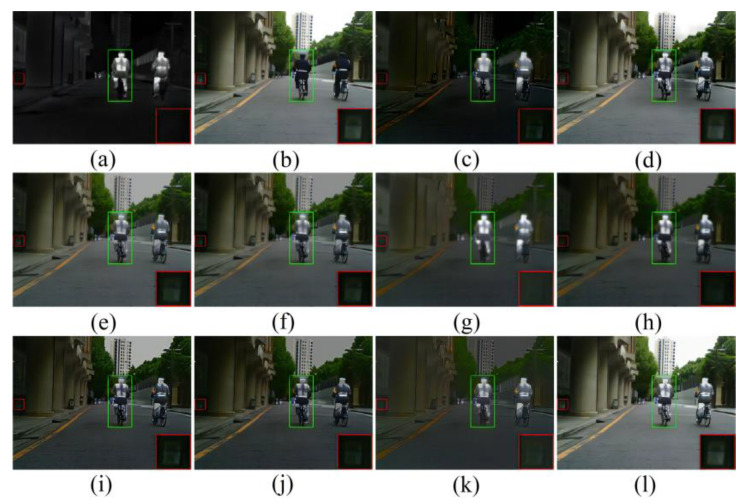
Comparison of 10 image fusion methods in daytime scenes. (**a**) Infrared. (**b**) Visible. (**c**) GTF. (**d**) MST-SR. (**e**) DenseFuse. (**f**) RFN-Nest. (**g**) FusionGAN. (**h**) GANMcC (**i**) IFCNN. (**j**) U2Fusion. (**k**)SDNet. (**l**) SeAFusion. The green area is to highlight the fusion effect, while the red area is to highlight the fusion effect in the details.

As shown in Figure 10 [15], GTF and Fusion GAN did not preserve the texture details of the visible image, and Fusion GAN did not sharpen the edges of the highlighted object. Although Dense Fuse, RFN-Nest, GANMcC, U2Fusion, and SDNet fused the detailed information of the visible light image, and the thermal radiation information of the infrared image, they were inevitably disturbed by useless information in the fusion process. We zoomed in on an area with a red box, to illustrate the phenomenon that texture detail is distorted by the spectrum, to varying degrees. In addition, the highlighted parts with green boxes were used to show the problem that useless information weakens the highlighted targets. SeAFusion and MST-SR can preserve rich texture detail while highlighting objects; however, MST-SR is slightly polluted by thermal radiation information in some background areas, such as the ground in the images below. 

**Figure 11:**
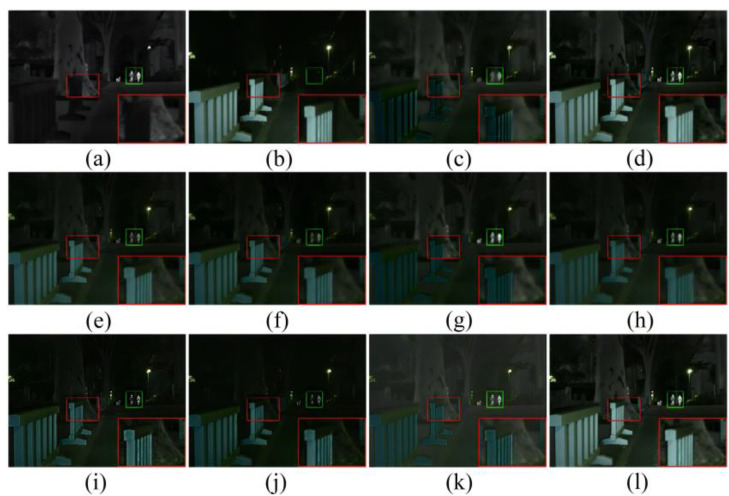
Comparison of 10 image fusion methods in a night scene. (**a**) Infrared. (**b**) Visible. (**c**) GTF. (**d**) MST-SR. (**e**) DenseFuse. (**f**) RFN-Nest. (**g**) FusionGAN. (**h**) GANMcC (**i**) IFCNN. (**j**) U2Fusion. (**k**)SDNet. (**l**) SeAFusion. The green area is to highlight the fusion effect, while the red area is to highlight the fusion effect in the details.

In night scenes, infrared and visible images can only provide limited scene information; therefore, it is a challenge to adaptively integrate valuable information from infrared and visible images. As shown in Figure 11, all algorithms fuse the complementary information in infrared and visible images to some extent, but there are still some subtle changes in the fusion results of different algorithms. In particular, GTF and Fusion GAN blur the contour of the thermal radiation target, and the texture range of GTF is seriously polluted by the spectrum. In addition to SeAFusion, other methods introduce some useless information into the fusion image, mainly manifested in the contamination of detail textures and the weakening of prominent objects.

### 5.2. Quantitative Results

In order to quantitatively compare the fusion results of different fusion algorithms, we selected six typical fusion image evaluation metrics, namely EN, MI, VIF, SF, SD, and Qabf. EN can reflect the amount of information in the fused image—usually, the larger the EN, the richer the information from the fused image; MI maximally indicates that this method transfers the information from the source image to the fused image; VIF indicates that the fused image more closely matches the human visual system; SF reflects the rate of change of the image grayscale—the larger the spatial frequency means the clearer the image, and the better the quality of the fused image; SD reflects the discrete degree of a set of values—the larger the SD, the more obvious the image edge information; Qabf reflects the quality of the visual information obtained from the input image fusion. 

**Figure 12:**
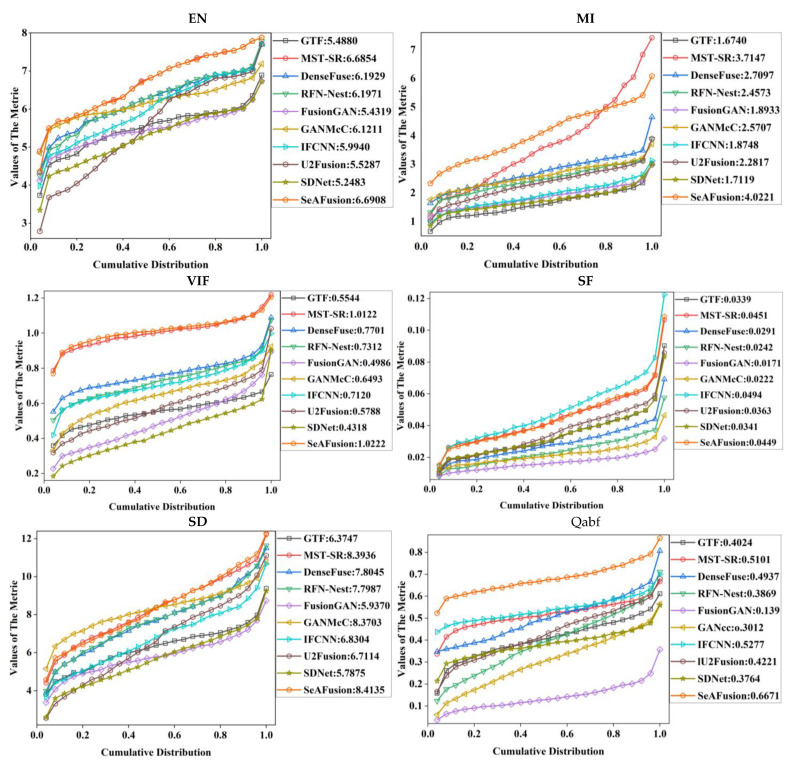
Quantitative comparison of EN, MI, VIF, SF, SD, and Qabf indicators for 361 image pairs from the MFNet dataset.

As shown in Figure 12, among the 10 typical image fusion algorithms, SeAFusion showed unique advantages in EN, MI, VIF, and Qabf. The algorithm obtained the largest amount of information, which was transferred from the source image to the fused image. The algorithm was more consistent with the human visual system and retained more edge information. Thanks to the powerful fine-grained feature extraction capability of GRDB, the contrast of the fused image was good. IFCNN and MST-SR performed better in SF metrics.

## 6. Future Prospects for Image Fusion Technology 

Image fusion techniques have evolved from traditional algorithms, such as multiscale fusion and sparse matrices, to deep learning algorithms, and the introduction of deep learning tools has provided a significant boost to various tasks of image fusion. In particular, based on data-driven hierarchical feature representation and end-to-end model training, the corresponding deep models exhibit significant performance gains over traditional methods. In recent years, some new network structures and training techniques have further enriched deep learning theory, and continue to inject new energy into the field of image fusion; however, the research of image fusion algorithms based on deep learning still faces some challenges. This paper provides an outlook on image fusion techniques, anticipating that scholars in the field will make important breakthroughs in these directions:

(1) From the perspective of dataset generation. How to clearly and explicitly define the target and background of different fusion images is a prerequisite for large-scale fair comparison of fusion algorithms. In the data acquisition, the alignment accuracy of the infrared and visible images in the actual scene is improved, and the spatial transformation is set as a variable factor to synchronize the alignment and fusion, to reduce the artifacts. To reduce the noise of infrared images, we can introduce feature detection algorithms to extract the main infrared targets and minimize noise interference. We can also design a multi-aperture imaging system to segment the acquired infrared images, so as to improve the image resolution and expand the field of view.

(2) From the perspective of neural network construction. A promising direction is to design lightweight, interpretable, and well-generalized model components. The existing mainstream frameworks are divided into two strategies: early fusion and late fusion. The optimal fusion strategies for different application scenarios deserve in-depth study. Meanwhile, in the process of exploring a neural network to implement multiple fusion tasks, reasonable parameter sharing, parameter isolation, and different fusion tasks can promote each other instead of limiting each other.

(3) From the perspective of loss function design. An urgent question to be verified is whether the final loss function, obtained by weighted summation of different loss functions, which is heavily used at this stage, is perceptually relevant to human vision or machine vision. Meanwhile, the design of perceptually relevant loss functions with good mathematical properties and low computational complexity is a top priority in the field of image fusion.

(4) From the perspective of evaluation metrics. As the existing evaluation metrics cannot truly and effectively reflect the perceptual performance of fusion algorithms, how to efficiently and objectively perform subjective quality evaluation needs to be re-emphasized.

(5) From the perspective of the application area. Different fusion methods can be combined and innovated according to the characteristics of different scenarios; however, the performance of the algorithms needs to be considered comprehensively when building hybrid models. In order to meet engineering applications, the real-time performance of fusion algorithms needs to be improved, and parallel computing (the process of using multiple computing resources simultaneously to solve computational problems) applied to the field of image fusion, to realize the parallelism of algorithms in time and space, and to improve the operation efficiency.

## 7. Conclusions

Image fusion of infrared and visible light is a popular area of information fusion research, which has been developing rapidly in recent years. In this paper, we have summarized the commonly used infrared and visible image fusion methods from both traditional image fusion (including multiscale transform, sparse representation, and subspace) and deep learning image fusion (including AE, CNN, and GAN). We have also summarized the hybrid image fusion methods, from practical engineering considerations, to solve the practical multi-scene and multi-target image fusion problems. In addition, we have presented the applications of infrared and visible image fusion methods to robot vision, medical imaging, agricultural remote sensing, and industrial defect detection. In order to measure the advantages and disadvantages of image fusion methods, we have summarized the commonly used image fusion evaluation indexes, in terms of subjective evaluation and objective evaluation. We have then selected classical data sets and typical image fusion evaluation methods for testing, and have presented the results graphically. Finally, we have given an outlook on the development of image fusion. We expect that scholars will quickly grasp the current status and development trend of the image fusion field by reading this paper and advance the development of image fusion technology based on the preceding work.

## Figures and Tables

**Table 1:** Advantages and disadvantages of multiscale decomposition methods.

Methods	Typical Method	Advantages	Disadvantages
Pyramid transformation	Laplace pyramid transformation; ratio low-pass pyramid [32]; contrast pyramid [33]; morphological pyramid [34]; controllable pyramid [35].	Opens up the basic idea of multiscale transform pixel-level image fusion research with simple implementation and fast operation speed.	Non-directional, sensitive to noise, not stable when reconstructed, and redundant between pyramid layers.
Wavelet transform [36,37]	Discrete wavelet transform (DWT), dual-tree discrete wavelet transform (DTWT), and lifting wavelet transform (DWT).	Good time-frequency localization, directionality, no redundancy, and high utilization of image frequency band information.	Does not have direction selectivity and translation invariance, and is weak in extracting the edge information of the image.
Multiscale geometric analysis [38]	Non-subsampled contourlet transform (NSCT); non-subsampled shear wave transform (NSST).	The frequency localization, multi-directionality, high variance and sparsity of the image can be better approximated and described.	Does not have translational invariance, and is prone to pseudo-Gibbs phenomenon near the singularities, and high computational complexity.

**Table 2:** Typical evaluation indices and mathematical models.

Evaluation Index	Mathematical Models and Explanations	
EN [90]	$\mathrm{EN}=-\sum_{n=0}^{N-1}p_{n}\log_{2}p_{n}$	(11)
	$\mathrm{where}\;N\;\mathrm{represents}\;\mathrm{the}\;\mathrm{gray}\;\mathrm{level}\;\mathrm{of}\;\mathrm{the}\;\mathrm{fused}\;\mathrm{image}\;\mathrm{and}\;p_{n}$ represents the normalized histogram of the corresponding gray level of the fused image.	
MI [4]	$\mathrm{MI}=\sum_{i=0}^{L-1}\sum_{j=0}^{L-1}P_{AB}(i,j)\log_{2}\frac{P_{AB}(i,j)}{P_{A}(i)P_{B}(j)}$	(12)
	$\mathrm{where}\;P_{AB}(i,j)$$\;\mathrm{is}\;\mathrm{the}\;\mathrm{normalized}\;\mathrm{joint}\;\mathrm{probability}\;\mathrm{density}\;\mathrm{distribution}\;\mathrm{between}\;\mathrm{source}\;\mathrm{image}\;A\;\mathrm{and}\;\mathrm{fused}\;\mathrm{image}\;B,\;P_{A}(i)$$,\;P_{B}(j)$ is the histogram statistical probability of images *A* and *B*, respectively, and *L* is the number of gray levels.	
VIF [91]	$\mathrm{VIF}=\frac{\sum_{k\in \;\mathrm{sub}\text{-}\mathrm{band}}\sum_{b}I\left(C_{b},F_{b}\right)}{\sum_{k\in \;\mathrm{sub}\text{-}\mathrm{band}}\sum_{b}I\left(C_{b},E_{b}\right)}=\frac{\sum_{k}\sum_{b}\log_{2}\left(1+\frac{g_{k,b}^{2}s_{k,b}^{2}C_{u}}{\left(\sigma_{v_{k,b}}^{2}+\sigma_{N}^{2}\right)I}\right)}{\sum_{k}\sum_{b}\log_{2}\left(1+\frac{s_{k,b}^{2}C_{u}}{\sigma_{N}^{2}I}\right)}$	(13)
	$\mathrm{where}\;k\;\mathrm{and}\;b\;\mathrm{stand}\;\mathrm{for}\;\mathrm{sub}\text{-}\mathrm{band}\;\mathrm{and}\;\mathrm{block}\;(RF)\;\mathrm{index},\;\mathrm{respectively};\;g_{k,b}$$\;\mathrm{is}\;\mathrm{the}\;\mathrm{scalar}\;\mathrm{gain}\;\mathrm{field}\;\mathrm{in}\;\mathrm{the}\;b\mathrm{th}\;\mathrm{block}\;\mathrm{at}\;\mathrm{the}\;k\mathrm{th}\;\mathrm{sub}\text{-}\mathrm{band},\;\mathrm{and}\;s_{k,b}$$,\;\mathrm{and}\;C_{u}$$\;\mathrm{are}\;\mathrm{defined}\;\mathrm{correspondingly}.\;\mathrm{It}\;\mathrm{is}\;\mathrm{evident}\;\mathrm{that}\;g_{k,b}$$\;\mathrm{and}\;s_{k,b}$$,\;\mathrm{are}\;\mathrm{generalized}\;\mathrm{definitions}\;\mathrm{of}\;g_{i}$$\;\mathrm{and}\;s_{i}$ when considering multiple sub-bands.	
SF [92]	$\mathrm{SF}=\sqrt{RF^{2}+CF^{2}}$	(14)
	$\mathrm{where}\;RF=\frac{1}{M\times N}\sum_{i=1}^{M-1}\sum_{j=1}^{N-1}{\left[F(i,j)-F(i,j+1)\right]}^{2}$$\;\mathrm{and}\;CF=\frac{1}{M\times N}\sum_{i=1}^{N-1}\sum_{j=1}^{M-1}{\left[F(i,j)-F(i+1,j)\right]}^{2}$ are the row and column frequencies of the image.	
SD [93]	$\mathrm{SD}=\frac{1}{MN}\sqrt{\sum_{i=1}^{M}\sum_{j=1}^{N}{\left(F(i,j)-\mu \right)}^{2}}$	(15)
	where *M*, *N* are the width and height of the image, μ is the mean value, and *F* is the pixel value of the image at position *i*, *j*.	
Qabf [94]	$Q(a,b,f)=\frac{1}{\left|W\right|}\sum_{w\in W}\left(\lambda (w)Q_{0}(a,f|w)+(1-\lambda (w))Q_{0}(b,f|w)\right)$	(16)
	$\mathrm{where}\;\lambda (w)=\frac{s(a|w)}{s(a|w)+s(b|w)}$$,\;Q_{0}(a,b)=\frac{1}{\left|W\right|}\sum_{w\in W}Q_{0}(a,b|w)$W is the family of all windows, and |W| is the cardinality of W.	

## Data Availability

No new data were created in this manuscript.

## References

[1] Li G., Xie H., Yan W., Chang Y., Qu X. (2020). Detection of Road Objects with Small Appearance in Images for Autonomous Driving in Various Traffic Situations Using a Deep Learning Based Approach. IEEE Access.

[2] Liu Y., Chen X., Wang Z., Wang Z.J., Ward R.K., Wang X. (2017). Deep learning for pixel-level image fusion: Recent advances and future prospects. Inf. Fusion.

[3] Li S., Kang X., Fang L., Hu J., Yin H. (2016). Pixel-level image fusion: A survey of the state of the art. Inf. Fusion.

[4] Ma J., Ma Y., Li C. (2019). Infrared and visible image fusion methods and applications: A survey. Inf. Fusion.

[5] El-Gamal F.E.Z.A., Elmogy M., Atwan A. (2016). Current trends in medical image registration and fusion. Egypt. Inform. J..

[6] Ma J., Chen C., Li C., Huang J. (2016). Infrared and visible image fusion via gradient transfer and total variation minimization. Inf. Fusion.

[7] Liu Y., Liu S., Wang Z. (2014). A general framework for image fusion based on multi-scale transform and sparse representation. Inf. Fusion.

[8] Li H., Wu X.J. (2018). DenseFuse: A fusion approach to infrared and visible images. IEEE Trans. Image Process..

[9] Li H., Wu X.J., Kittler J. (2021). RFN-Nest: An end-to-end residual fusion network for infrared and visible images. Inf. Fusion.

[10] Ma J., Yu W., Liang P., Li C., Jiang J. (2019). FusionGAN: A generative adversarial network for infrared and visible image fusion. Inf. Fusion.

[11] Ma J., Zhang H., Shao Z., Liang P., Xu H. (2020). GANMcC: A Generative Adversarial Network with Multiclassification Constraints for Infrared and Visible Image Fusion. IEEE Trans. Instrum. Meas..

[12] Zhang Y., Liu Y., Sun P., Yan H., Zhao X., Zhang L. (2020). IFCNN: A general image fusion framework based on convolutional neural network. Inf. Fusion.

[13] Zhu C., Zeng M., Huang X. (2018). SDnet: Contextualized attention-based deep network for conversational question answering. arXiv.

[14] Xu H., Ma J., Jiang J., Guo X., Ling H. (2020). U2Fusion: A unified unsupervised image fusion network. IEEE Trans. Pattern Anal. Mach. Intell..

[15] Tang L., Yuan J., Ma J. (2022). Image fusion in the loop of high-level vision tasks: A semantic-aware real-time infrared and visible image fusion network. Inf. Fusion.

[16] Huang B., Yang F., Yin M., Mo X., Zhong C. (2020). A review of multimodal medical image fusion techniques. Comput. Math. Methods Med..

[17] Pure A.A., Gupta N., Shrivastava M. (2013). An overview of different image fusion methods for medical applications. Int. J. Sci. Eng. Res..

[18] Du J., Li W., Lu K., Xiao B. (2016). An overview of multi-modal medical image fusion. Neurocomputing.

[19] Hermessi H., Mourali O., Zagrouba E. (2021). Multimodal medical image fusion review: Theoretical background and recent advances. Signal Process..

[20] Yang Y., Han C., Kang X., Han D. An overview on pixel-level image fusion in remote sensing. Proceedings of the 2007 IEEE International Conference on Automation and Logistics.

[21] Pohl C., van Genderen J. (2014). Remote sensing image fusion: An update in the context of Digital Earth. Int. J. Digit. Earth.

[22] Belgiu M., Stein A. (2019). Spatiotemporal Image Fusion in Remote Sensing. Remote Sens..

[23] Wang Q., Yu D., Shen Y. An overview of image fusion metrics. Proceedings of the 2009 IEEE Instrumentation and Measurement Technology Conference.

[24] Omar Z., Stathaki T. Image fusion: An overview. Proceedings of the 2014 5th International Conference on Intelligent Systems, Modelling and Simulation.

[25] Liu Y., Chen X., Liu A., Ward R.K., Wang Z.J. (2021). Recent Advances in Sparse Representation Based Medical Image Fusion. IEEE Instrum. Meas. Mag..

[26] Burt P.J., Adelson E.H. (1987). The Laplacian pyramid as a compact image code. Readings in Computer Vision.

[27] Liu Y., Jin J., Wang Q., Shen Y., Dong X. (2014). Region level based multi-focus image fusion using quaternion wavelet and normalized cut. Signal Process..

[28] Liu X., Mei W., Du H. (2017). Structure tensor and nonsubsampled shearlet transform based algorithm for CT and MRI image fusion. Neurocomputing.

[29] Zhang Q., Maldague X. (2016). An adaptive fusion approach for infrared and visible images based on NSCT and compressed sensing. Infrared Phys. Technol..

[30] Li H., Wu X.-J., Kittler J. (2020). MDLatLRR: A Novel Decomposition Method for Infrared and Visible Image Fusion. IEEE Trans. Image Process..

[31] Liu Y., Chen X., Ward R.K., Wang Z.J. (2016). Image Fusion with Convolutional Sparse Representation. IEEE Signal Process. Lett..

[32] Toet A. (1989). Image fusion by a ratio of low-pass pyramid. Pattern Recognit. Lett..

[33] Toet A., Van Ruyven L.J., Valeton J.M. (1989). Merging thermal and visual images by a contrast pyramid. Opt. Eng..

[34] Toet A. (1989). A morphological pyramidal image decomposition. Pattern Recognit. Lett..

[35] Freeman W.T., Adelson E.H. (1991). The design and use of steerable filters. IEEE Trans. Pattern Anal. Mach. Intell..

[36] Grossmann A., Morlet J. (1984). Decomposition of Hardy Functions into Square Integrable Wavelets of Constant Shape. SIAM J. Math. Anal..

[37] Mallat S.G. (1989). A theory for multiresolution signal decomposition: The wavelet representation. IEEE Trans. Pattern Anal. Mach. Intell..

[38] Da Cunha A.L., Zhou J., Do M.N. (2006). The Non-subsampled Contourlet Transform: Theory, Design, and Applications. IEEE Trans. Image Process..

[39] Yu X., Ren J., Chen Q., Sui X. (2014). A false color image fusion method based on multi-resolution color transfer in normalization YCBCR space. Optik.

[40] Jin H., Jiao L., Liu F., Qi Y. (2008). Fusion of infrared and visual images based on contrast pyramid directional filter banks using clonal selection optimizing. Opt. Eng..

[41] Zhang B. Study on image fusion based on different fusion rules of wavelet transform. Proceedings of the 2010 3rd International Conference on Advanced Computer Theory and Engineering (ICACTE).

[42] Selesnick I.W., Baraniuk R.G., Kingsbury N.C. (2005). The dual-tree complex wavelet transform. IEEE Signal Process. Mag..

[43] Zou Y., Liang X., Wang T. (2013). Visible and infrared image fusion using the lifting wavelet. Telkomnika Indones. J. Electr. Eng..

[44] Yin S., Cao L., Tan Q., Jin G. Infrared and visible image fusion based on NSCT and fuzzy logic. Proceedings of the 2010 IEEE International Conference on Mechatronics and Automation.

[45] Liu H.X., Zhu T.H., Zhao J.J. (2013). Infrared and visible image fusion based on region of interest detection and nonsubsampled contourlet transform. J. Shanghai Jiaotong Univ. (Sci.).

[46] Guo K., Labate D. (2007). Optimally Sparse Multidimensional Representation Using Shearlets. SIAM J. Math. Anal..

[47] Kong W., Lei Y., Zhao H. (2014). Adaptive fusion method of visible light and infrared images based on non-subsampled shearlet transform and fast non-negative matrix factorization. Infrared Phys. Technol..

[48] Bin Y., Shutao L. (2010). Multifocus Image Fusion and Restoration with Sparse Representation. IEEE Trans. Instrum. Meas..

[49] Rubinstein R., Zibulevsky M., Elad M. (2010). Double Sparsity: Learning Sparse Dictionaries for Sparse Signal Approximation. IEEE Trans. Signal Process..

[50] Zhang Q., Liu Y., Blum R.S., Han J., Tao D. (2018). Sparse representation based multi-sensor image fusion for multi-focus and multi-modality images: A review. Inf. Fusion.

[51] Biswas C., Ganguly D., Mukherjee P.S., Bhattacharya U., Hou Y. (2022). Privacy-aware supervised classification: An informative subspace based multi-objective approach. Pattern Recognit..

[52] Fu Z., Wang X., Xu J., Zhou N., Zhao Y. (2016). Infrared and visible images fusion based on RPCA and NSCT. Infrared Phys. Technol..

[53] Cvejic N., Bull D., Canagarajah N. (2007). Region-Based Multimodal Image Fusion Using ICA Bases. IEEE Sensors J..

[54] Ma J., Tang L., Fan F., Huang J., Mei X., Ma Y. (2022). SwinFusion: Cross-domain long-range learning for general image fusion via swin transformer. IEEE/CAA J. Autom. Sin..

[55] Granato D., Santos J.S., Escher G.B., Ferreira B.L., Maggio R.M. (2018). Use of principal component analysis (PCA) and hierarchical cluster analysis (HCA) for multivariate association between bioactive compounds and functional properties in foods: A critical perspective. Trends Food Sci. Technol..

[56] Baviristti D.P., Dhuli R. (2016). Two-scale image fusion of visible and infrared images using saliency detection. Infrared Phys. Technol..

[57] Cvejic N., Lewis J., Bull D., Canagarajah N. Adaptive Region-Based Multimodal Image Fusion Using ICA Bases. Proceedings of the 2006 9th International Conference on Information Fusion.

[58] Song H.A., Lee S.Y. (2013). Hierarchical Representation Using NMF. International Conference on Neural Information Processing.

[59] Mou J., Gao W., Song Z. Image fusion based on non-negative matrix factorization and infrared feature extraction. Proceedings of the 2013 6th International Congress on Image and Signal Processing.

[60] Hao S., He T., An B., Ma X., Wen H., Wang F. (2022). VDFEFuse: A novel fusion approach to infrared and visible images. Infrared Phys. Technol..

[61] Li H., Wu X.-J., Durrani T. (2020). NestFuse: An Infrared and Visible Image Fusion Architecture Based on Nest Connection and Spatial/Channel Attention Models. IEEE Trans. Instrum. Meas..

[62] Xu H., Zhang H., Ma J. (2021). Classification Saliency-Based Rule for Visible and Infrared Image Fusion. IEEE Trans. Comput. Imaging.

[63] Liu Y., Chen X., Cheng J., Peng H. A medical image fusion method based on convolutional neural networks. Proceedings of the 2017 20th International Conference on Information Fusion.

[64] Zhang H., Xu H., Xiao Y., Guo X., Ma J. (2020). Rethinking the Image Fusion: A Fast Unified Image Fusion Network based on Proportional Maintenance of Gradient and Intensity. Proc. Conf. AAAI Artif. Intell..

[65] Zhang H., Ma J. (2021). SDNet: A Versatile Squeeze-and-Decomposition Network for Real-Time Image Fusion. Int. J. Comput. Vis..

[66] Ma J., Tang L., Xu M., Zhang H., Xiao G. (2021). STDFusionNet: An Infrared and Visible Image Fusion Network Based on Salient Target Detection. IEEE Trans. Instrum. Meas..

[67] Zhu J.Y., Park T., Isola P., Efros A.A. Unpaired Image-to-Image Translation Using Cycle-Consistent Adversarial Networks. Proceedings of the 2017 IEEE International Conference on Computer Vision (ICCV).

[68] Choi Y., Choi M., Kim M., Ha J.W., Kim S., Choo J. Stargan: Unified generative adversarial networks for multi-domain image-to-image translation. Proceedings of the IEEE Conference on Computer Vision and Pattern Recognition.

[69] Xu H., Liang P., Yu W., Jiang J., Ma J. Learning a Generative Model for Fusing Infrared and Visible Images via Conditional Generative Adversarial Network with Dual Discriminators. Proceedings of the Twenty-Eighth International Joint Conference on Artificial Intelligence.

[70] Ma J., Liang P., Yu W., Chen C., Guo X., Wu J., Jiang J. (2020). Infrared and visible image fusion via detail preserving adversarial learning. Inf. Fusion.

[71] Ma J., Xu H., Jiang J., Mei X., Zhang X.-P. (2020). DDcGAN: A Dual-Discriminator Conditional Generative Adversarial Network for Multi-Resolution Image Fusion. IEEE Trans. Image Process..

[72] Li J., Huo H., Li C., Wang R., Feng Q. (2021). AttentionFGAN: Infrared and Visible Image Fusion Using Attention-Based Generative Adversarial Networks. IEEE Trans. Multimed..

[73] Liu Z., Feng Y., Chen H., Jiao L. (2017). A fusion algorithm for infrared and visible based on guided filtering and phase congruency in NSST domain. Opt. Lasers Eng..

[74] Meng F., Song M., Guo B., Shi R., Shan D. (2017). Image fusion based on object region detection and Non-Subsampled Contourlet Transform. Comput. Electr. Eng..

[75] Zhang B., Lu X., Pei H., Zhao Y. (2015). A fusion algorithm for infrared and visible images based on saliency analysis and non-subsampled Shearlet transform. Infrared Phys. Technol..

[76] Cai J., Cheng Q., Peng M., Song Y. (2017). Fusion of infrared and visible images based on nonsubsampled contourlet transform and sparse K-SVD dictionary learning. Infrared Phys. Technol..

[77] Yin M., Duan P., Liu W., Liang X. (2017). A novel infrared and visible image fusion algorithm based on shift-invariant dual-tree complex shearlet transform and sparse representation. Neurocomputing.

[78] Majumder B.D., Roy J.K., Padhee S. (2018). Recent advances in multifunctional sensing technology on a perspective of multi-sensor system: A review. IEEE Sens. J..

[79] Kaur H., Koundal D., Kadyan V. (2021). Image Fusion Techniques: A Survey. Arch. Comput. Methods Eng..

[80] Chen Y., Peng X., Kong L., Dong G., Remani A., Leach R. (2021). Defect inspection technologies for additive manufacturing. Int. J. Extrem. Manuf..

[81] Chen Y., Duan T., Wang C., Zhang Y., Huang M. (2021). End-to-End Ship Detection in SAR Images for Complex Scenes Based on Deep CNNs. J. Sensors.

[82] Martínez S.S., Vázquez C.O., García J.G., Ortega J.G. (2017). Quality inspection of machined metal parts using an image fusion technique. Measurement.

[83] Chan A.L., Schnelle S.R. (2013). Fusing concurrent visible and infrared videos for improved tracking performance. Opt. Eng..

[84] Piella G. (2003). A general framework for multiresolution image fusion: From pixels to regions. Inf. Fusion.

[85] Toet A., IJspeert J.K., Waxman A.M., Aguilar M. (1997). Fusion of visible and thermal imagery improves situational awareness. Displays.

[86] Toet A., Franken E.M. (2003). Perceptual evaluation of different image fusion schemes. Displays.

[87] Tsai Y., Lee Y., Matsuyama E. (2008). Information entropy measure for evaluation of image quality. J. Digit. Imaging.

[88] Sheikh R., Bovik C. (2006). Image information and visual quality. IEEE Trans. Image Process..

[89] Petrovic S., Xydeas S. (2004). Gradient-based multiresolution image fusion. IEEE Trans. Image Process..

[90] Van Aardt J. (2008). Assessment of image fusion procedures using entropy, image quality, and multispectral classification. J. Appl. Remote Sens..

[91] Han Y., Cai Y., Cao Y., Xu X. (2013). A new image fusion performance metric based on visual information fidelity. Inf. Fusion.

[92] Petrovic V., Xydeas C. Objective image fusion performance characterization. Proceedings of the Tenth IEEE International Conference on Computer Vision (ICCV’05).

[93] Zhu X.X., Bamler R. (2013). A Sparse Image Fusion Algorithm with Application to Pan-Sharpening. IEEE Trans. Geosci. Remote Sens..

[94] Piella G., Heijmans H. A new quality metric for image fusion. Proceedings of the 2003 International Conference on Image Processing (Cat. No 03CH37429).

